# Shell-breaking predation on gastropods by *Badister pictus* (Coleoptera, Carabidae) with strikingly asymmetric mandibles

**DOI:** 10.3897/zookeys.1044.62293

**Published:** 2021-06-16

**Authors:** Masakazu Hayashi, Shinji Sugiura

**Affiliations:** 1 Hoshizaki Green Foundation, Sono, Izumo, 691-0076, Japan Hoshizaki Green Foundation Izumo Japan; 2 Graduate School of Agricultural Science, Kobe University, 1-1 Rokkodai, Nada-ku, Kobe 657–8501, Japan Kobe University Kobe Japan

**Keywords:** Anti-predator defense, *
Badister
*, freshwater snail, Gastropoda, land snail, Licinini

## Abstract

The adults and larvae of some groups in the coleopteran family Carabidae are known to prey on snails (Mollusca: Gastropoda). Most species of the carabid tribe Licinini are believed to feed on live snails. However, the snail-eating behavior of only a few species has been studied. Whether adults of the licinine genus *Badister* can prey on live snails was tested by providing 155 live snails of 20 species (eleven terrestrial and nine aquatic species) to adults of *Badister pictus* Bates, 1873, and observing their behavior under laboratory conditions. Six of the 20 snail species have an operculum that can close the aperture of the shell. Each *B. pictus* adult attacked all of the snails provided. *Badister pictus* successfully preyed on ten terrestrial and six aquatic snail species. These beetles used their strikingly asymmetrical mandibles to break the dextral shells along the dorsal part of the whorls from the outer lip of the aperture towards the apex, allowing subsequent consumption of the soft bodies. However, 41.9% of snails could not be eaten by *B. pictus* adults. The rate of predation success by *B. pictus* decreased with increasing shell size and thickness of snails. In addition, the presence of an operculum decreased the rate of predation success by *B. pictus*. The results show that the shell size, thickness, and operculum of some snail species could play important roles in preventing *B. pictus* mandibles from breaking the shells. Therefore, *Badister* beetles may exert selective pressure on the evolution of defensive shell structures in small-sized snails.

## Introduction

Gastropods protect their soft bodies from predators through shells (Vermeij 1974); however, some gastropod-eating animals use specific structures and tactics to break open these shells to allow effective access to the bodies (Zipser and Vermeij 1978; Palmer 1979; Inoda et al. 2003; Konuma and Chiba 2007). For example, crabs can crush hard shells using their strong claws (Zipser and Vermeij 1978), whereas fish can also crush shells with their jaws (Palmer 1979). Larvae of giant water scavenger beetles use their asymmetrical mandibles to break the right-coiled shells of some freshwater snail species (Inoda et al. 2003; Hayashi and Sugiura 2020). However, some gastropod species have developed further defensive armor and structures to protect themselves from specialized predators (Vermeij 1976).

In the coleopteran family Carabidae, adults and larvae of some groups are snail hunters (Sturani 1962; Digweed 1993; Arnett and Thomas 2000; Konuma and Chiba 2007; Riddick 2008; Erwin et al. 2015). In particular, most species of the carabid tribe Licinini are believed to prey on live snails (Arnett and Thomas 2000; Erwin et al. 2015). For example, adults of the genera *Licinus* Latreille, 1802 and *Diplocheila* Brullé, 1834 break snail shells using their extraordinarily asymmetrical mandibles (El-Moursy and Ball 1959; Brandmayr and Zetto Brandmayr 1986; Ball 1992). Although Licinini comprises 238 species in 23 genera (Anichtchenko et. al. 2021), the snail-eating behavior has been studied in only a few species (Brandmayr and Zetto Brandmayr 1986).

The licinine genus *Badister* Clairville, 1806, which comprises 48 species, is found in all zoogeographical regions (Arnett and Thomas 2000; Anichtchenko et. al. 2021). *Badister* beetles inhabit open land habitats, including lakeshores, riverbanks, and grasslands (Arnett and Thomas 2000; Erwin and Ball 2011; Tamutis et al. 2018). *Badister* adults have asymmetrical mandibles that are morphologically similar to those of *Licinus* (Ball 1992; Arnett and Thomas 2000), and so *Badister* adults have been predicted to be snail feeders (Arnett and Thomas 2000). The late Dr. Terry Erwin and his colleague previously described a new species of *Badister* from South America (Erwin and Ball 2011). In addition, Erwin et al. (2015) hypothesized that almost all species of the Licinini, including *Badister*, are snail hunters. Because the adults of *Badister* (body length < 10 mm) are smaller than those of *Licinus* (> 10 mm; Erwin and Ball 2011), the snail-eating behavior of *Badister* adults may be different from that of *Licinus* adults; however, these hypotheses have not yet been tested. In the present study, we provided various types of snails to adults of *Badister pictus* Bates, 1873 under laboratory conditions to investigate whether *B. pictus* can prey on live snails using their mandibles. Furthermore, we investigated the effects of shell morphology on the predation success of *B. pictus*. Finally, we discuss the importance of *Badister* adults as snail-eating specialists and the defensive function of shell morphology in small-sized snails.

## Materials and methods

### Study species

*Badister pictus* is found in the wet grasslands along paddy fields, lakeshores, and riverbanks of Japan, Taiwan, and Russia (Morita 2001; Yahiro 2007; Mori 2018; Anichtchenko et al. 2021). Four males and seven females of *B. pictus* (body length as mean ± standard error: 6.9 ± 0.1; range: 6.5–7.5 mm) were collected using light traps at Sono-cho (35°26'53"N, 132°51'58"E, alt. 3 m), Izumo City, Shimane Prefecture, Honshu, Japan between 20 July and 28 August 2020. This site is located in Shinji-ko Green Park, which contains trees and man-made ponds, and is surrounded by paddy fields and irrigation channels. The park is also adjacent to the western coast of the brackish water lake Shinji-ko. Terrestrial and aquatic snails that were abundant in the study site were collected from several sites in the eastern Shimane Prefecture (35°10–16'N, 132°33–51'E) between the same dates. A total of 155 juveniles and adults of 20 snail species (maximum shell height or width < 13 mm) were used for feeding experiments with the *B. pictus* adults (Table 1). Eleven of the snail species were terrestrial and nine were aquatic. Nineteen of the species had dextral (right coiled) shells and one had sinistral (left coiled) shells (Table 1). The ratio of shell height to width ranged from 0.2 to 3.4 (Table 1). Six species have an operculum that can close the aperture of the shell (Table 1). All snails were identified based on shell morphology (Azuma 1982, 1995; Masuda and Uchiyama 2010) and distribution information (Hayashi et al. 2016; Mashino and Kawano 2018, 2019).

The specimens of *B. pictus* and snail shells (except broken shells) examined in this study were deposited at the Hoshizaki Institute for Wildlife Protection (**HOWP**), Izumo, Japan.

**Table 1.** Gastropod species and predation by *Badister pictus* observed in this study.

Family	Species	Habitat*	Eaten by beetles			Shell size and shape			
			%	*N*	Stage†	Size‡	H/W§	Chirality	Operculum
Achatinidae	*Opeas pyrgula* Schmacker & Boettger, 1891	Ter	91.7	12	J, A	2.4–7.7	2.0–3.4	Dextral	Absent
Alycaeidae	*Metalycaeus hirasei* (Pilsbry, 1900)	Ter	0.0	10	A	3.8–4.3	0.6–0.7	Dextral	Present
Assimineidae	*Assiminea japonica* Martens, 1877	Aqu	0.0	4	J	5.0–5.4	1.1–1.5	Dextral	Present
Camaenidae	*Aegista aemula* (Gude, 1900)	Ter	100.0	1	J	6.5	0.6	Dextral	Absent
	*Euhadra dixoni* (Pilsbry, 1900)	Ter	33.3	3	J	6.4–8.4	0.7–0.8	Dextral	Absent
	*Euhadra idzumonis* (Pilsbry & Gulock, 1900)	Ter	100.0	11	J	3.1–3.9	0.6–0.8	Dextral	Absent
	*Euhadra subnimbosa* (Kobelt, 1894)	Ter	100.0	1	J	5.6	0.8	Dextral	Absent
Cyclophoridae	*Cyclophorus herklotsi* Martens, 1860	Ter	22.2	9	J	4.2–7.0	0.8–1.0	Dextral	Present
Diplommatinidae	*Diplommatina* sp.	Ter	20.0	10	J, A	3.1–4.5	1.7–2.0	Dextral	Absent
Euconulidae	*Yamatochlamys* sp.	Ter	100.0	1	A	3.7	0.6	Dextral	Absent
Gastrodontidae	*Zonitoides arboreus* (Say, 1816)	Ter	100.0	14	J, A	2.8–4.4	0.4–0.6	Dextral	Absent
Lymnaeidae	*Pseudosuccinea columella* (Say, 1817)	Aqu	58.8	17	J, A	5.9–12.5	1.6–1.8	Dextral	Absent
Physidae	*Physa acuta* Draparnaud, 1805	Aqu	80.0	15	J, A	3.8–8.1	1.6–1.9	Sinistral	Absent
Planorbidae	*Gyraulus tokyoensis* (Mori, 1938)	Aqu	100.0	1	A	3.9	0.2	Dextral	Absent
	*Hippeutis cantori* (Benson, 1850)	Aqu	100.0	15	J, A	3.8–7.7	0.2–0.4	Dextral	Absent
	*Polypylis hemisphaerula* (Benson, 1842)	Aqu	100.0	2	A	4.4–4.9	0.3–0.5	Dextral	Absent
Semisulcospiridae	*Semisulcospira libertina* (Gould, 1859)	Aqu	40.0	10	J	5.0–7.0	1.5–2.0	Dextral	Present
Stenothyridae	*Stenothyra japonica* Kuroda, 1962	Aqu	0.0	12	A	4.1–4.7	1.5–1.9	Dextral	Present
Diapheridae	*Sinoennea iwakawa* (Pilsbry, 1900)	Ter	66.7	3	A	3.1–3.2	1.8–1.9	Dextral	Absent
Viviparidae	*Sinotaia quadrata histrica* (Gould, 1859)	Aqu	0.0	4	J	6.2–6.3	1.1–1.1	Dextral	Present

* Ter, terrestrial; Aqu, aquatic. † J, juveniles; A, adults. ‡ Range of maximum shell height or width (mm). § Ratios of maximum shell height/maximum shell width.

### Observation of beetle mandibles

A stereomicroscope (SMZ-1000, Nikon, Tokyo, Japan) with a CCD camera unit (Digital Sight, DS-L2, Nikon, Tokyo, Japan) and a scanning electron microscope (JCM-6000 Neoscope; JEOL Ltd., Tokyo, Japan) were used to observe and photograph the mandibles of *B. pictus*. The photographs taken using the stereomicroscope were stacked using Adobe Photoshop CS2 for Macintosh. The samples used for scanning electron microscope (SEM) observations were dehydrated and gold-coated using high-vacuum evaporation (Smart Coater, DII-2910SCTR, JEOL Ltd., Tokyo, Japan). The morphology of the mandibles, especially the asymmetry of the left and right mandibles, was investigated. The terminology for mandibular morphology follows Ball (1992).

### Feeding experiments

Adults of *B. pictus* were individually placed in plastic containers (size W 129 × D 99 × H 60 mm, capacity 500 mL) with wet paper (Kimwipe S-200: Nippon Paper Crecia Co. Ltd., Tokyo) under laboratory conditions (mean temperature, 29.1 °C; range: 25–31 °C) between 25 July and 2 September 2020. The feeding behavior of each *B. pictus* adult was observed by the unaided eye in a well-lit laboratory during the daytime, as described below.

A live snail was placed within the field of view of a single *B. pictus* in its plastic container. Terrestrial and aquatic snails were provided to *B. pictus* under the same conditions. When the *B. pictus* adult attacked the snail, feeding behavior was observed. If the *B. pictus* adult could not successfully prey on the snail, another snail was provided. However, no *B. pictus* adult was fed more than one snail per day. Beetles were starved for 22–29 h before the feeding experiments. Snails with soft bodies that were not injured were considered to have survived, even if the shell or operculum were partially damaged. Adults of *B. pictus* were repeatedly used in feeding experiments (mean ± SE 14.1 ± 4.8 snails exposed per adult beetle), although individual snails were used only once. The raw data are available from the Figshare Digital Repository (https://figshare.com/s/89fdf111feea86bb8626).

### Measurement of shell morphology

Shell morphological characteristics, such as shell size and thickness, were examined to clarify how these factors affected the ability of *B. pictus* adults to open them. The sizes of the shells were measured from the images taken using a Canon Eos70D (Canon Inc., Tokyo, Japan) with a macro lens (MP-E 65 mm; Canon Inc., Tokyo, Japan) at equal magnification. Each image was magnified 115 times and measured using the digital image processing software Preview ver. 10.1 (Apple Inc.). The maximum height and width of the shells were measured to the closest 0.1 mm. The aperture thickness of some shells (63 shells of 14 species) was also measured (Fig. 4) to the closest 1 µm, under SEM. The data are available from the Figshare Digital Repository (https://figshare.com/s/89fdf111feea86bb8626).

### Data analysis

Generalized linear mixed models (GLMMs) with a binomial error distribution and a logit link were used to elucidate the factors affecting successful predation of snails (R software version 2.15.3 with the lme4 package 0.999999-0; Bates et al. 2012). Success of predation on a snail or not (1/0) was coded as a binary response variable. The shell size (maximum height/width) and operculum presence/absence of each snail were treated as fixed factors. Beetle individuals and snail species were treated as random effects. Because the GLMM analysis showed that the interaction between shell size and operculum presence/absence was not significant (*P* > 0.05; Suppl. material 2: Table S1), this interaction was not included in the final fixed factor model. Curves were fitted using logistic regression, based on the GLMM results. Similarly, the effects of shell thickness on predation success, coded as a binary variable as above, were analyzed using GLMM. The thickness of the shell aperture and operculum presence/absence of each snail were treated as fixed factors. Snail species was treated as a random effect.

## Results

### Morphology of beetle mandibles

**Figure 1.**
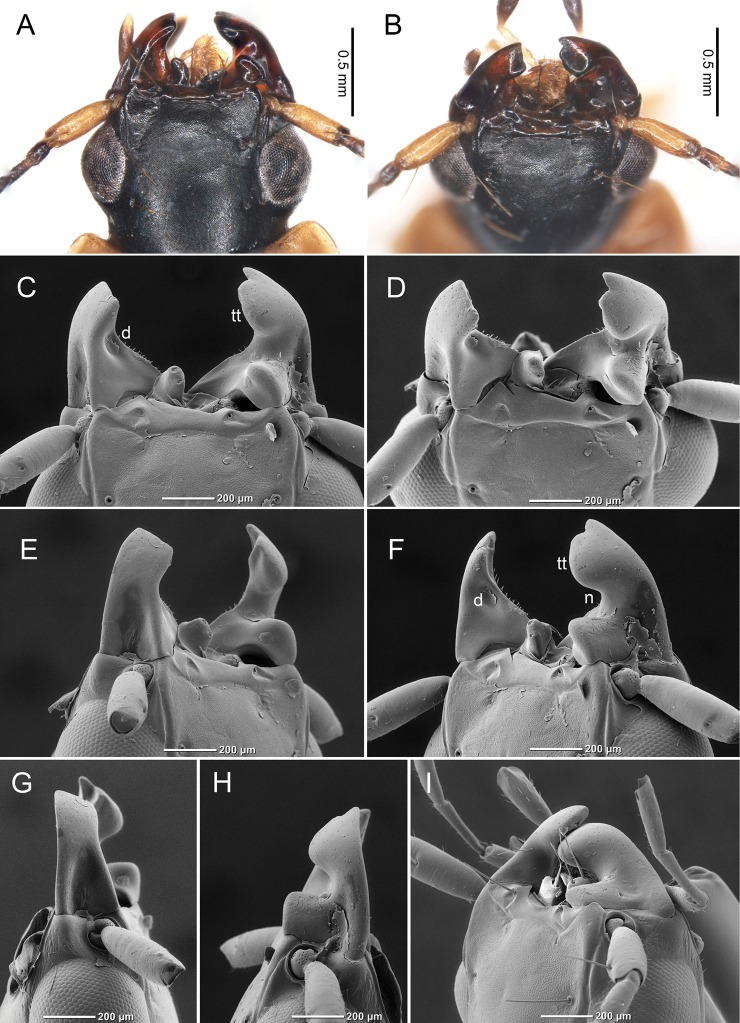
Mandibles of adult *Badister pictus***A, B** photos from a stereoscopic microscope **C–I** photos by a SEM**A, C** dorsal view **B, D** dorsal view (from the front) **E** dorsal view (from the left mandible) **F** dorsal view (from the right mandible) **G** lateral view (from the left mandible) **H** lateral view (from the right mandible) **I** dorsal view of closed mandibles (from the right mandible). “d” indicates a depression of the left mandible; “n” indicates a dorsal notch of the right mandible; “tt” indicates a terebral tooth of the right mandible. All antennal segments (except the first segment) and all setae on labrum were removed in **C–H**.

Similar to other *Badister* species, *B. pictus* had clearly asymmetric mandibles (Fig. 1A–I) and labrum (Fig. 1A). The left mandible was truncated (Fig. 1), and the inner side of the left mandible was depressed (Fig. 1C, F). The right mandible had a dorsal notch (Fig. 1F), and the tip of the right mandible had a semicircular blade (i.e., a terebral tooth; Fig. 1F) with a small tooth underneath. When the left and right mandibles were closed, the projection of the right mandible fit the depression of the left mandible (Fig. 1I).

### Feeding behavior

**Figure 2.**
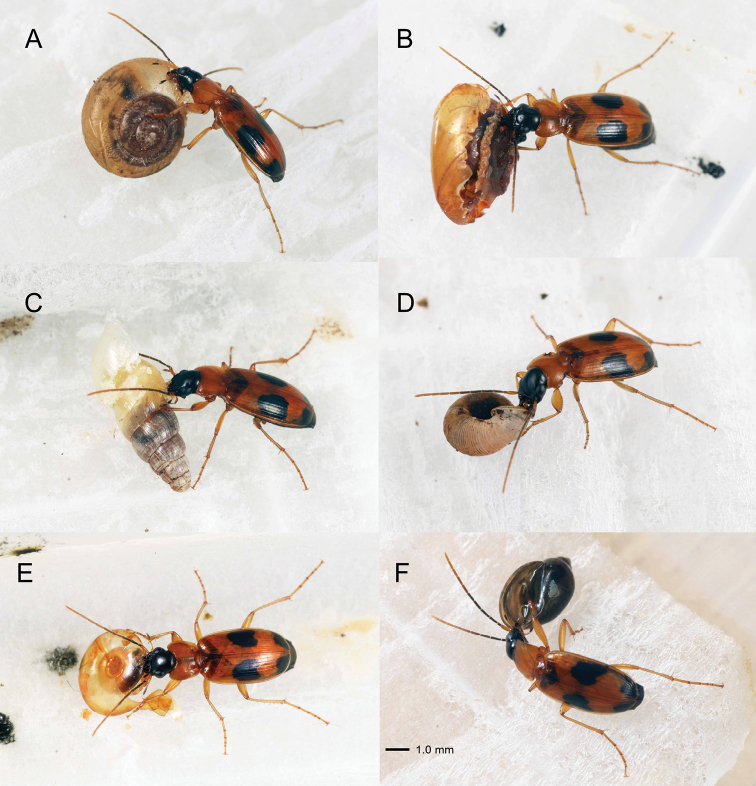
Attacking and feeding behavior of *Badister pictus* adults **A** adult biting the outer lip of *Zonitoides arboreus* (Say, 1816) **B** adult feeding on the soft body after opening the shell of *Z. arboreus***C** adult breaking the shell of *Opeas pyrgula* Schmacker & Boettger, 1891 **D** adult biting the outer lip of *Metalycaeus hirasei* (Pilsbry, 1900) **E** adult feeding on the soft body from the broken shell of *Hippeutis cantori* (Benson, 1850) **F** adult biting the basal lip of the sinistral snail *Physa acuta* Draparnaud, 1805.

**Figure 3.**
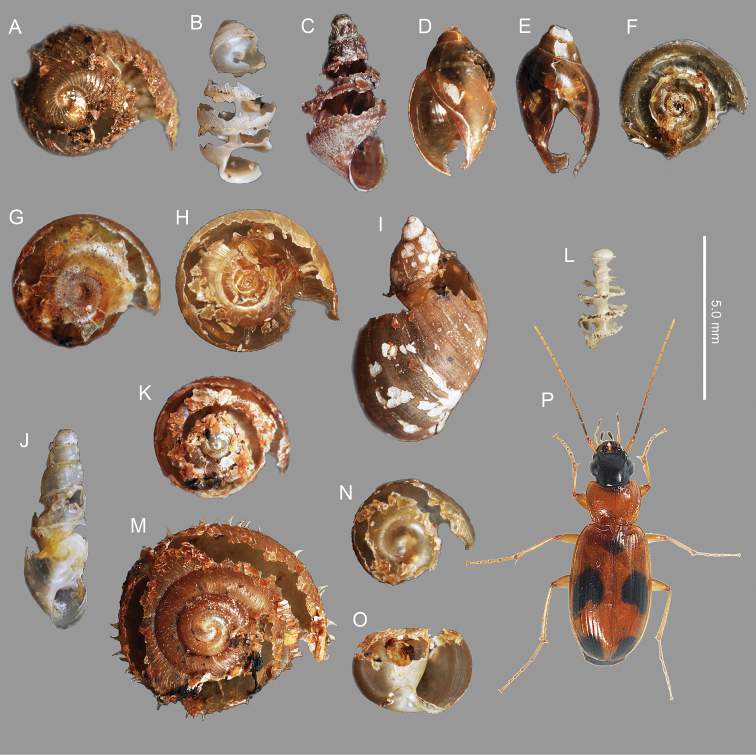
Shells broken by *Badister pictus***A***Cyclophorus herklotsi* Martens, 1860 **B***Diplommatina* sp. **C***Semisulcospira libertina* (Gould, 1859) **D, E***Physa acuta***F***Gyraulus tokyoensis* (Mori, 1938) **G***Hippeutis cantori***H***Polypylis hemisphaerula* (Benson, 1842) **I***Pseudosuccinea columella* (Say, 1817) **J***Opeas pyrgula***K***Zonitoides arboreus***L***Sinoennea iwakawa* (Pilsbry, 1900) **M***Aegista aemula* (Gude, 1900) **N, O***Euhadra idzumonis* (Pilsbry & Gulock, 1900) **P** adult *B. pictus***A, F, G, H, K, M, N** dorsal view **B, C, D, J, L, O** front view **E, I** back view. The soft bodies of these snails were eaten by *B. pictus*.

All adults of *B. pictus* always attacked the provided snails. Of the 75 terrestrial snails (11 species), 61.3% (10 species) were eaten by *B. pictus*, whereas 55.0% of 80 aquatic snails (six of nine species) were eaten (Table 1; Figs 2, 3).

**Figure 4.**
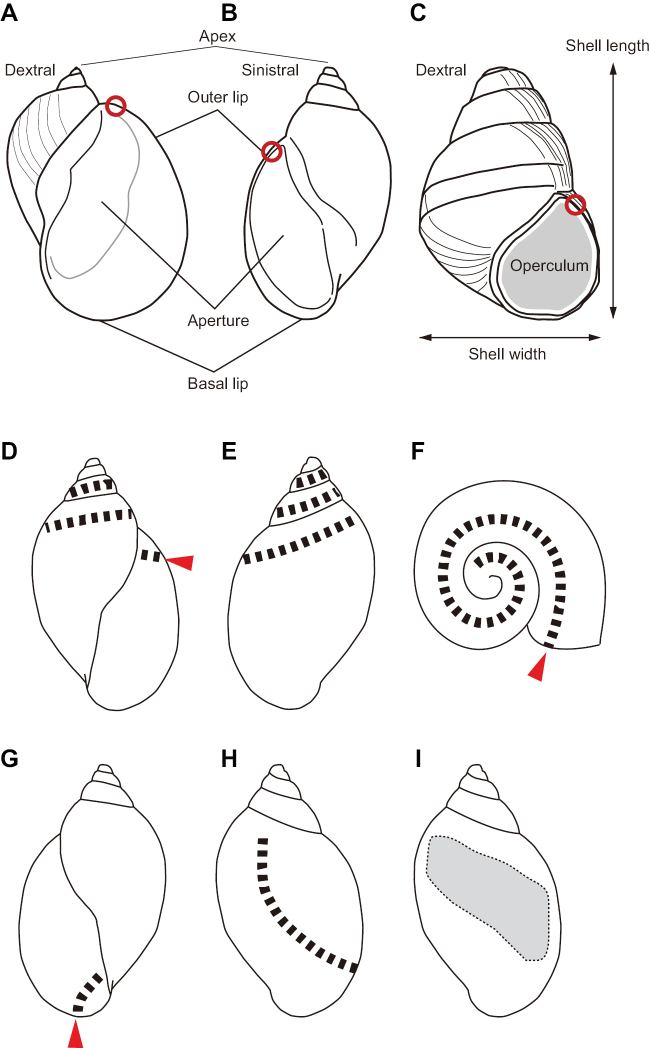
Shell morphology and bite traces of *Badister pictus***A** dextral shell **B** sinistral shell **C** dextral shell with an operculum **D–F** bite traces on dextral shells **G, H** bite traces on sinistral shells **I** broken part (shaded area) on a sinistral shell **A–D, G** front view **E, H, I** back view **F** dorsal view. Red circles indicate the positions where the shell (aperture) thickness was measured. Red arrows indicate the starting point of shell breaking by *B. pictus*. Broken lines indicate the bite traces by *B. pictus*.

**Figure 5.**
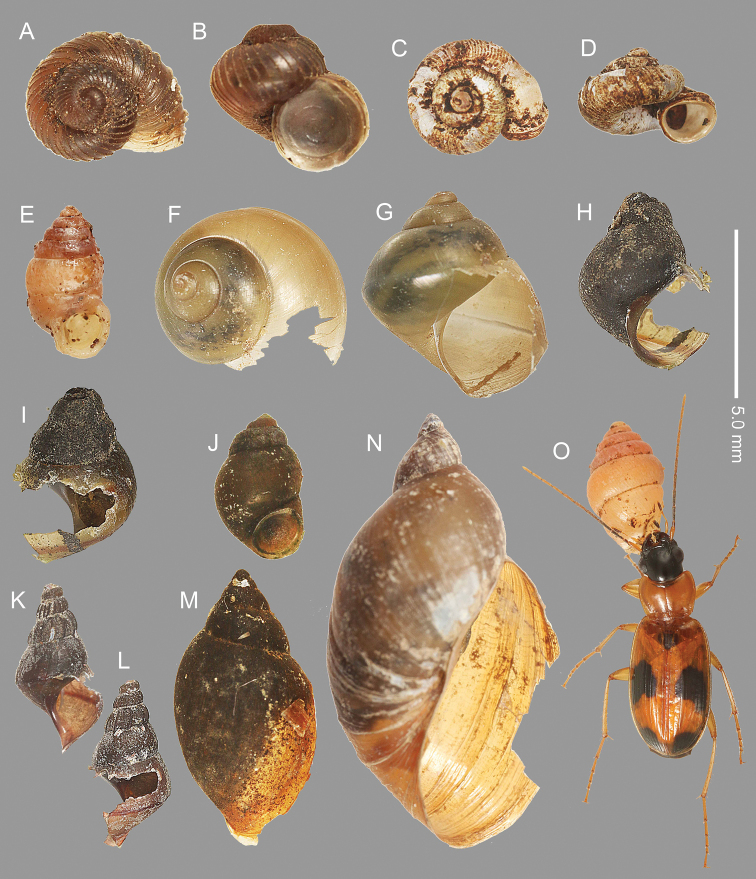
Shells of snails surviving attack by *Badister pictus***A, B***Cyclophorus herklotsi***C, D***Metalycaeus hirasei***E***Diplommatina* sp., 1904 **F, G***Sinotaia quadrata histrica* (Gould, 1859) **H, I***Assiminea japonica* Martens, 1877 **J***Stenothyra japonica* Kuroda, 1962 **K, L***Semisulcospira libertina***M***Physa acuta***N***Pseudosuccinea columella***O** adult *B. pictus* attacking *Diplommatina* sp. **A, C, F** dorsal view **B, D, E, G, H, J, K, N** front view **I, L, M** back view. The soft bodies were not eaten by *B. pictus*, although some shells were partially broken.

Beetle adults always began their attacks by breaking the outer lip of the dextral (right-coiled) shells (Figs 2A, 4D; see Suppl. material 1: Movie 1), and the left and right mandibles were always placed against the external and internal shell walls, respectively (Fig. 2A). When the outer lip of each shell was cracked by biting, the beetles used the mandibles to break open the shells along the dorsal part of the whorls towards the apex (Figs 2B, C, 3, 4D–F). Although attacked snails retracted their bodies into the unopened shells to avoid the beetles’ mandibles, the beetles were often able to insert both the left and right mandibles into the opened shells to feed on the snail’s body (Fig. 2B, E). Small-sized shells (3.1–3.2 mm) could be broken right up to the apex (Fig. 3L). Some adults of *B. pictus* gave up attacking snails without successful predation. Consequently, 41.9% of the snails survived these attacks (Table 1; Fig. 5).

### Effects of shell morphology

**Figure 6.**
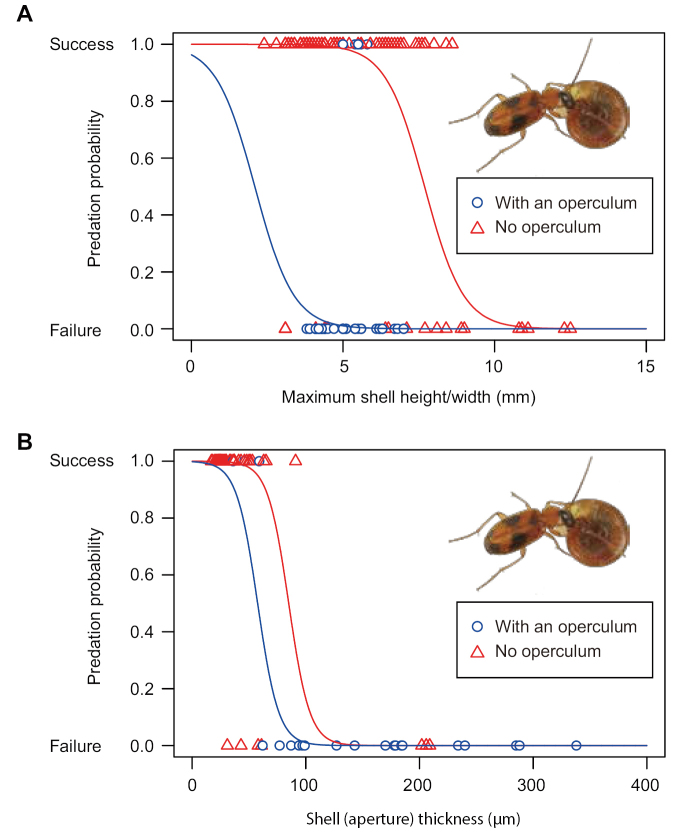
Effects of shell size/thickness and operculum on predation success of *Badister pictus***A** relationship between maximum shell height/width and the presence/absence of an operculum and predation success (*N* = 155) **B** relationship between the shell thickness and the presence of an operculum and the predation success (*N* = 55). Red triangles and blue circles show snails with and without an operculum, respectively. Red and blue lines represent logistic regression lines of snails with and without an operculum derived from generalized linear mixed models, respectively (Tables 2, 3).

The rate of successful predation on snails by *B. pictus* decreased with increasing shell size (Fig. 6A). In addition, the presence of an operculum decreased the rate of predation success (Fig. 6A). The GLMM indicated that the effects of shell size and presence of an operculum were significant factors in determining predation (Table 2). Similarly, the rate of predation success by *B. pictus* decreased with increasing shell aperture thickness (Fig. 6B); adults of *B. pictus* thus could not successfully prey on snails with thick apertures (> 100 µm; Fig. 2D; Table 3). However, the presence of an operculum did not significantly influence the rate of predation success of *B. pictus* (Table 3).

Feeding on sinistral (left-coiled) snails (*Physa acuta* Draparnaud, 1805) by *B. pictus* was also observed. All juveniles (*N* = 12) of *P. acuta* were eaten by *B. pictus*, whereas all adults (*N* = 3) of *P. acuta* survived the attack. *Badister pictus* adults started breaking the basal lip (i.e., the opposite side of the outer lip) or the shell wall from the exterior side of the body whorl of *P. acuta* (Figs 2F, 4G–I), although they always started breaking the outer lip of dextral (right-coiled) snails (Figs 2A, 4D–F). Apparently, because the basal lip of adult *P. acuta* was thicker than that of the juveniles, *B. pictus* could not break the sinistral shells of adult *P. acuta* (Fig. 5M).

**Table 2.** Results of a generalized linear mixed model investigating the effects of shell size and operculum presence on predation by *Badister pictus*.

Response variable	Explanatory variable	Coefficient estimate	SE	*z* value	*P* value
	(fixed effect)				
Predation success	Intercept	11.986	2.603	4.605	0.000004
	Shell size	–1.559	0.377	–4.135	0.000036
	Operculum presence*	–8.725	2.477	–3.523	0.000427

* Operculum absence was used as a reference.

**Table 3.** Results of a generalized linear mixed model investigating the effects of shell thickness and operculum presence on predation by *Badister pictus*.

Response variable	Explanatory variable	Coefficient estimate	SE	*z* value	*P* value
	(fixed effect)				
Predation success	Intercept	9.1669	3.0543	3.001	0.00269
	Shell thickness	–0.1079	0.0446	–2.418	0.01559
	Operculum presence*	–2.9508	2.6212	–1.126	0.26028

* Operculum absence was used as a reference.

## Discussion

Most species of the tribe Licinini are believed to be snail hunters (Arnett and Thomas 2000; Erwin et al. 2015). In particular, adults of *Badister* have been predicted to feed on snails using their mandibles (Arnett and Thomas 2000). In this study, we have demonstrated that, similar to other licinine species (Brandmayr and Zetto Brandmayr 1986; Ball 1992), *B. pictus* has clearly asymmetric mandibles and labrum (Fig. 1) and that they can use the mandibles to break snail shells as required for successful predation (Table 1; Figs 2–4). Therefore, our observations support the hypothesis that adults of *Badister* use their mandibles to prey on snails. Furthermore, our results indicate that the shell morphologies and opercula of some snails likely play important roles in defending against *B. pictus* predation.

*Badister pictus* is found in wet grasslands, such as paddy fields, in Japan (Yahiro 2007; Mori 2018), and thus occupies habitats in which snails occur. Although the feeding ecology and prey items have not been investigated under field conditions, our laboratory experiments suggest that adults of *B. pictus* respond to snails as potential prey and that they are capable of successful predation on both terrestrial and aquatic snails. Aquatic snails of the families Lymnaeidae and Physidae that crawl out of water to escape from underwater predators (Hayashi and Sugiura 2020) can potentially be attacked by terrestrial predators, including *B. pictus*. Because adults of another licinine species, *Diplocheila oregona* (Hatch, 1951), reportedly preyed on aquatic snails under laboratory conditions (El-Moursy and Ball 1959), licinine beetles could be an important predator of aquatic snails as well as terrestrial ones.

The mandibles and snail-eating behavior of *B. pictus* observed in this study were similar to those of *Licinus* adults reported in a previous study (Brandmayr and Zetto Brandmayr 1986). However, attack on snails with an operculum by *B. pictus* was different from that observed in *Licinus cassideus* (Fabricius, 1792). When the aperture of a provided snail was closed by a hard calcareous operculum, *L. cassideus* started crushing the shell from the exterior side of the body whorl, not far from the aperture (Brandmayr and Zetto Brandmayr 1986). Therefore, *L. cassideus* could successfully prey on snails with an operculum. However, some *B. pictus* individuals failed to attack snails with an operculum (Table 1), possibly because the operculum and thick body whorl prevented *B. pictus* from breaking the shells using their mandible (Fig. 5). These differences in predatory success may reflect differences in body and mandible size between *B. pictus* (body length 6.5–7.5 mm) and *L. cassideus* (> 15 mm).

As most terrestrial snails have dextral shells, licinine beetles most often encounter right-coiled prey. Brandmayr and Zetto Brandmayr (1986) hypothesized that the asymmetrical mandibles of licinine beetles are an adaptation to preying on dextral snails. Comparison of feeding on dextral and sinistral snails could be conducted to test this hypothesis (cf. Inoda et al. 2003; Hoso et al. 2007). In the present study, *B. pictus* adults showed the same attack behavior in various types of dextral snails: they always started breaking the outer lip of the dextral snails, and thereafter broke the shells along the dorsal part of the whorls towards the apex (Figs 2, 3; Suppl. material 1: Movie 1). If attacking sinistral snails (*P. acuta*), *B. pictus* adults began by breaking the basal lip, but not the outer lip. Consequently, *B. pictus* could not successfully break (open) the sinistral shells along the dorsal part of the whorls from the lip (Figs 4, 5). These results suggest that the mandibles and prey feeding behavior of *B. pictus* are adapted to preying on dextral snails. The extreme asymmetry between mandibles is a common feature of the tribe Licinina; some species have a dorsal notch of the right mandible, whereas others have a notch of the left mandible (Ball 1992). How the left-right asymmetry of carabid mandibles plays an important role in eating dextral snails remains unclear. Further experiments are required explore this topic.

Diverse shell morphologies of gastropods have been discussed in terms of anti-predator defenses (Vermeij 1976; Hoso and Hori 2008; Wada and Chiba 2013; Liew and Schilthuizen 2014; Vermeij 2015; Sato 2019; Němec and Horsák 2019). Although previous studies have focused on the defensive traits of relatively large-sized snails (Vermeij 1975; Hoso and Hori 2008; Vermeij 2015; Sato 2019), only a few studies have investigated the defensive roles of shell morphologies and other traits in small-sized snails (shell size < 10 mm; Wada and Chiba 2013; Liew and Schilthuizen 2014). Our results showed that shell thickness and operculum presence in some small-sized snail species could play important roles in defending against the small-sized carabid species *B. pictus* (body length 6.5–7.5 mm). Large-sized carabids such as *Damaster blaptoides* Kollar, 1836 (> 30 mm), would not have influenced the evolution of shell morphologies in small-sized snails, because they could easily crush any type of small-sized shells with their large mandibles (Konuma and Chiba 2007). However, our results suggest that small-sized carabids such as *Badister* beetles, exert selective pressure on the evolution of defensive morphologies in small-sized snails.

## Supplementary materials

Supplementary material 1 Movie 1. *Badister pictus* attacking a dextral snail *Zonitoides arboreus*. Data type images Explanation note Beetle adults always started breaking the outer lip of the dextral (right-coiling) shells. https://binary.pensoft.net/file/555544This dataset is made available under the Open Database License (http://opendatacommons.org/licenses/odbl/1.0/). The Open Database License (ODbL) is a license agreement intended to allow users to freely share, modify, and use this Dataset while maintaining this same freedom for others, provided that the original source and author(s) are credited.Masakazu Hayashi, Shinji Sugiura

Supplementary material 2 Table S1. Results of a generalized linear mixed model for effects of shell size, operculum presence, and the interaction on predation by *Badister pictus*. Data type Table Explanation note Results of a generalized linear mixed model. https://binary.pensoft.net/file/555545This dataset is made available under the Open Database License (http://opendatacommons.org/licenses/odbl/1.0/). The Open Database License (ODbL) is a license agreement intended to allow users to freely share, modify, and use this Dataset while maintaining this same freedom for others, provided that the original source and author(s) are credited.Masakazu Hayashi, Shinji Sugiura
