# Supplementary material for: Shell-breaking predation on gastropods by Badister pictus (Coleoptera, Carabidae) with strikingly asymmetric mandibles
Source: Zookeys. 2021 Jun 16;1044:815–30. doi: 10.3897/zookeys.1044.62293 (PMC8222264; doi:10.3897/zookeys.1044.62293)
Supplement: Supplementary material 2 — Table S1. Results of a generalized linear mixed model for effects of shell size, operculum presence, and the interaction on predation by Badister pictus. [file zookeys-1044-815-s002.pdf]

Table S1. Results of a generalized linear mixed model for effects of shell size, operculum presence, and the interaction on predation by *Badister pictus*.

Response variable	Explanatory variable (fixed effect)	Coefficient estimate	SE	z value	p value
Predation success	Intercept	13.0045	2.9918	4.347	0.0000138
	Shell size	−1.7308	0.4456	−3.884	0.000103
	Operculum presence*	−13.8379	5.6763	−2.438	0.014776
	Shell size × operculum presence	0.9346	0.9555	0.978	0.328031

* Operculum absence was used as a reference.
